# *Pasteurella multocida* bacteremia in a patient with septic arthritis

**DOI:** 10.1016/j.imj.2022.08.001

**Published:** 2022-08-05

**Authors:** Shikha Malhotra, Tung Phan

**Affiliations:** Department of Pathology, University of Pittsburgh, Pittsburgh, Pennsylvania, USA

**Keywords:** Bacteremia, Arthritis, *Pasteurella mulocida*, Total knee replacement

## Abstract

*Pasteurella multocida* is a common commensal microorganism found in the nasopharynx of domestic and wild animals. Humans acquire *P multocida* infection primarily through contact with animals or their mucous secretions. *P multocida* is infrequently encountered in clinical settings, and it is considered as a zoonotic pathogen. In this study, we present an interesting case of septic arthritis and bacteremia caused by *P multocida* in a 62-year-old patient. The patient was treated with surgical procedures and antibiotic therapy, which made significant improvement. This case study highlights the importance of *P multocida* in causing zoonotic infection in humans.

## 1 Introduction

The members of the genus *Pasteurella* belonging to the family *Pasteurellaceae* are small, nonmotile, non-spore-forming, gram-negative microorganisms [1]*.* While being common commensals found in the nasopharynx of domestic and wild animals, *Pasteurella* can cause a variety of diseases such as hemorrhagic septicemia in cattle, atrophic rhinitis in pigs, and fowl cholera in birds [2,3]. Pasteurellosis is used as a bacterial infection caused by *Pasteurella*. There are at least 30 different *Pasteurella* species (https://lpsn.dsmz.de/genus/pasteurella), and *Pasteurella multocida* is the most common cause of human pasteurellosis because of its association with the pharyngeal flora of domestic pets [4]. *P multocida* is a zoonotic agent, and its transmission to humans often occurs through a bite or scratch from cats and dogs or contact with their mucous secretions [5]. However, unusual cases of vertical transmission from infected mother to neonate were also reported [6]. Every year approximately 20%-50% of 1-2 million people in the United States, who are bitten or scratched by dogs and cats, are infected by *P multocida* [7]. While this bacterium is often known to be associated with skin and soft tissue infections, *P multocida* also causes a wide range of infectious diseases in humans, including bacteremia, urinary tract infections, meningitis, endocarditis, pneumonia, brain abscess, conjunctivitis and epiglottitis, but the number of reported cases are small [1,8]. Breen and colleagues had seen only 23 patients diagnosed with *P multocida* infection in a major teaching hospital laboratory over a 10-year period in Australia [9]. The majority of them (73.9%, 17 of 23) had wound infections following animal bites, 3 cases had respiratory infections, and there were one case of neonatal meningitis and associated maternal vaginal carriage of *P multocida* [9]. In this study, we present an interesting case of an isolated *P multocida* in blood, synovial fluid, synovial tissue and prosthesis from a patient who had symptoms of right knee septic arthritis.

## 2 Case presentation

**Fig. 1:**
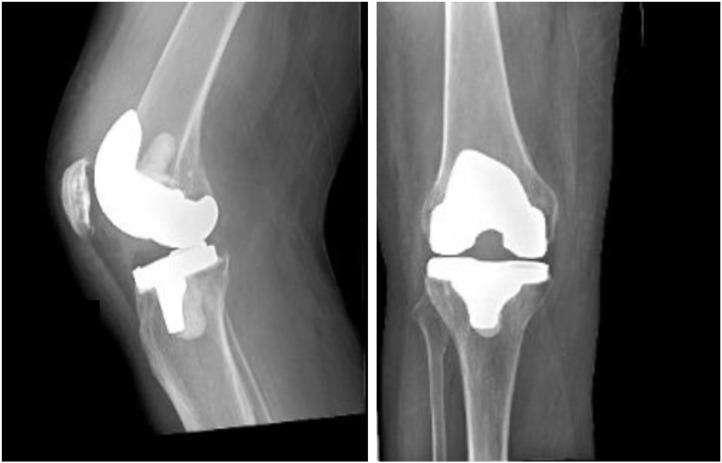
The preoperative radiograph showed the right total knee replacement with the prosthetic components.

**Fig. 2:**
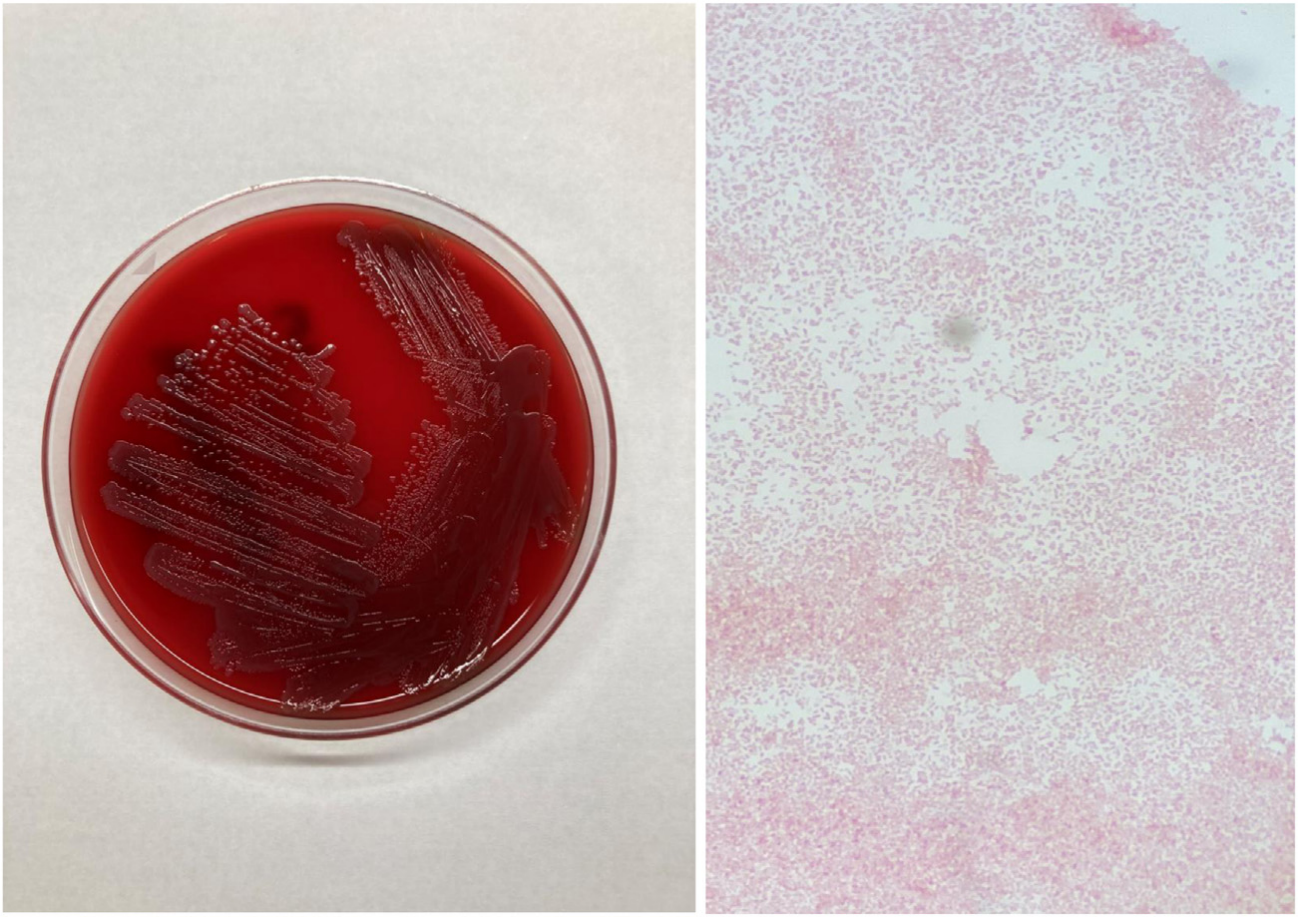
Grayish and non-hemolytic colonies of *Pasteurella multocida* were observed on sheep blood agar, and microscopic examination of a gram-stained smear revealed small gram-negative coccobacilli at 1000 x magnification.

A 62-year-old patient with a history of cirrhosis with esophageal varices, gastroesophageal reflux disease, hyperlipidemia and right knee arthroplasty (5 years ago) presented to the emergency room with acute onset of right knee pain. The patient stated that the swollen knee occurred over a week, and the patient was unable to bear weight on the right knee due to pain. Generalized weakness and low-grade fever developed over the last 2 days. Upon admission, the patient was febrile (38.3°C), but vitally stable. Physical examination revealed that the joint was warm to touch. Large joint effusion and decreased range of motion of the right knee were also noted on the physical examination. The X-ray showed the right total knee arthroplasty in a satisfactory alignment, and no osseous lesions were found (Fig. 1). Laboratory studies conducted at the time of admission revealed an elevated leukocytes in blood; the patient's white blood cell count was 14,000 per microliter with a neutrophilic predominance of 87%. Synovial fluid aspirate from the right knee had 115,000 white blood cells per microliter and neutrophils accounted for 86%. The patient was diagnosed with prosthetic joint infectious arthritis and was started empirically on ceftriaxone (2 g intravenously once) and vancomycin (1.5 g intravenously once). The next day, the patient underwent surgical debridement and washout procedures with a polyethylene liner exchange. After the surgery, the patient was placed on cefepime (2 g intravenously every 12 hours) and vancomycin (1.5 g intravenously every 12 hours) for 3 days. Synovial fluid, synovial tissue, blood and polyethylene liner were submitted to our clinical microbiology laboratory for bacterial culture. After 24 hours of incubation at 35°C in 5% CO_2_, there were grayish and nonhemolytic colonies on the nonselective blood and chocolate agar plates, which were planted from all submitted different specimen types. However, no growth was seen on the selective Columbia CNA and MacConkey agar plates. Small gram-negative coccobacilli were observed on the microscopic examination of a gram-stained smear (Fig. 2). The bacterial isolate was finally identified as *P multocida* by the MALDI-TOF system. Since its vancomycin resistance is common [8], vancomycin was discontinued, and the patient continued with cefepime (2 g intravenously every 12 hours) for additional 3 days. The patient was eventually discharged and did well on the one-month follow-up visit.

## 3 Discussion

*P multocida* was first discovered in 1878 and later named when this bacterium was found to be responsible for fowl disease in 1880 [2]. Since then, *P multocida* has been commonly found in domestic pets such as cats and dogs, but it is infrequently encountered in humans. *P multocida* infections in clinical settings often involve soft-tissue sites after animal bites or scratches. However, it can cause invasive diseases in immunocompromised patients or patients with chronic diseases [1,8]. Raffi and colleagues reported 13 cases of bacteremia caused by *P multocida* in a French general hospital during a 12-year period. The mortality rate was 31% and the majority (77%) of the patients had cirrhosis [10]. It is interesting that we had a rare case of *P multocida* bacteremia and septic arthritis in a patient with cirrhosis who had a unilateral total knee arthroplasty 5 years ago. Infection is one of serious complications of total joint arthroplasty [11]. While *P multocida* is an uncommon cause in prosthetic joint infections, other microorganisms such as *Staphylococcus aureus*, coagulase-negative staphylococci and *Streptococcus* species are widely responsible [12].

A vast majority of *P multocida* infection in humans is directly associated with a bite and/or scratch from dogs or cats. In the study, our patient lived with a dog and denied any bite or scratch from the dog. However, *P multocida* infection occasionally has been reported after exposure to cats or dogs in the absence of injury. Christenson et al. presented a case of fulminant *P multocida* sepsis in a renal transplant patient due to the cat's licking on the venous stasis ulcers on the patient's legs [13]. In 23 cases of *P multocida* meningitis in infants, 6 had known exposure without physical injury to dogs only, 4 to cats only, and 3 to both [14]. Honnorat and colleges retrospectively reviewed and identified 6 *P multocida* infected patients among 4686 cases of prosthetic joint infection over 20 years from 1993 to 2013 in southern France [15]. Among these, 2 cases had cat scratches, dog licking on surgical wound in few days before the beginning of symptoms was found in 2 cases, and an exposure with cats or dogs in 2 cases [15]. The finding in our patient was in line with these reports that a close contact with dogs and/or cats can pose a risk for *P multocida* infection. Taken together, our case study underscores the important role of a zoonotic pathogen *P multocida* in causing infectious diseases in humans.

## Funding

This research did not receive any specific grant from funding agencies in the public, commercial, or not-for-profit sectors.

## Author contributions

TP and SM: designed the study and wrote the manuscript.

## Acknowledgments

We thank the staff of the clinical microbiology laboratory at UPMC for help with initial isolation and characterization of the isolate.

## Declaration of competing interest

The authors declare no competing financial interests.

## Data available statement

The data that support the findings of this study are available on request from the corresponding author. The data are not publicly available due to privacy or ethical restrictions.

## Ethics statement

Approval from the ethical committee was not required due to the nature of this case report. Abiding by the Declaration of Helsinki, patient anonymity was guaranteed.

## Informed consent

Informed consent was waived for this study because no patients' data were reported.

## References

[1] Wilson A.B., Ho M. (2013). Pasteurella multocida: from zoonosis to cellular microbiology. Clin Microbiol Rev.

[2] Kubatzky K.F. (2012). Pasteurella multocida and immune cells. Curr Top Microbiol Immunol.

[3] Harper M., Boyce D.J., Adler B. (2006). Pasteurella multocida pathogenesis: 125 years after Pasteur. FEMS Microbiol Lett.

[4] Hurtado R., Maturrano L., Azevedo V. (2020). Pathogenomics insights for understanding Pasteurella multocida adaptation. Int J Med Microbiol.

[5] Kristinsson G. (2007). Pasteurella multocida infections. Pediatr Rev.

[6] Nakwan N., Nakwan N., Atta T. (2009). Neonatal pasteurellosis: a review of reported cases. Arch Dis Child Fetal Neonatal Ed.

[7] Iaria C., Cascio A. (2007). Please, do not forget Pasteurella multocida. Clin Infect Dis.

[8] Mogilner L., Katz C. (2019). Pasteurella multocida. Pediatr Rev.

[9] Breen D., Schonell M., Au T., Reiss-Levy E. (2000). Pasteurella multocida: a case report of bacteremic pneumonia and 10-year laboratory review. Pathology.

[10] Raffi F., Barrier J., Baron D. (1987). Pasteurella multocida bacteremia: report of thirteen cases over twelve years and review of the literature. Scand J Infect Dis.

[11] Tande J.A., Patel R. (2014). Prosthetic joint infection. Clin Microbiol Rev.

[12] Beam E., Osmon D. (2018). Prosthetic joint infection update. Infect Dis Clin North Am.

[13] Christenson E.S., Ahmed H.M., Durand C.M. (2015). Pasteurella multocida infection in solid organ transplantation. Lancet Infect Dis.

[14] Wade T., Booy R., Teare E.L. (1999). Pasteurella multocida meningitis in infancy - (a lick may be as bad as a bite). Eur J Pediatr.

[15] Honnorat E., Seng P., Savini H. (2016). Prosthetic joint infection caused by Pasteurella multocida: a case series and review of literature. BMC Infect Dis.

